# Aerosol-Derived Graphene Oxide Nanofilm Suppresses Adhesion-Dependent Survival and Migration in Pancreatic Ductal Adenocarcinoma Cells

**DOI:** 10.3390/ijms27104341

**Published:** 2026-05-13

**Authors:** Aleksandra Ciechońska, Mateusz Wierzbicki, Barbara Nasiłowska, Barbara Wójcik, Wojciech Skrzeczanowski, Katarzyna Ziółkowska, Marta Kutwin

**Affiliations:** 1 Faculty of Animal Breeding, Bioengineering and Conservation, Warsaw University of Life Sciences, 02-786 Warsaw, Poland; s213007@sggw.edu.pl; 2 Institute of Biology, Department of Nanobiotechnology, Warsaw University of Life Sciences, 02-786 Warsaw, Poland; mateusz_wierzbicki@sggw.edu.pl (M.W.); barbara_wojcik1@sggw.edu.pl (B.W.); 3 Biomedical Engineering Centre, Institute of Optoelectronics, Warsaw Military University of Technology, 00-908 Warsaw, Poland; barbara.nasilowska@wat.edu.pl (B.N.); wojciech.skrzeczanowski@wat.edu.pl (W.S.); 4 Faculty of Biology and Biotechnology, Warsaw University of Life Sciences, 02-776 Warsaw, Poland; s215129@sggw.edu.pl

**Keywords:** pancreatic adenocarcinoma, pancreatic cancer, BxPC-3, AsPC-1, graphene oxide aerosol, GO

## Abstract

Pancreatic ductal adenocarcinoma (PDAC) is the most aggressive malignancy, characterized by rapid progression, early metastasis, and resistance to conventional therapies. Increasing evidence indicates that the behavior of residual tumor cells is strongly influenced by physicochemical properties of their microenvironment. Surface engineering strategies using nanostructured materials may therefore represent a complementary approach to modulating cancer cell activity. In this study, we investigated whether a graphene oxide (GO) aerosol nanofilm modifies the biological behavior of PDAC cells in vitro. The GO aerosol (4.5 g/L) was characterized using STEM, DLS, zeta potential measurements, LIBS, EDX, and FTIR spectroscopy. Ultrastructural analysis revealed thin, wrinkled GO sheets forming partially overlapping lamellar structures, while physicochemical characterization confirmed a highly oxidized stable nanomaterial. Human PDAC cell lines (BxPC-3 and AsPC-1) were cultured on GO-modified substrates to assess morphology (SEM), metabolic activity (XTT assay), migratory capacity (wound healing assay over 72 h), and expression of genes related to proliferation and epithelial–mesenchymal transition (EMT) by RT-qPCR. GO nanofilm significantly reduced cell viability and inhibited migration in both cell lines. SEM analysis demonstrated shortened cytoplasmic projections and altered membrane integrity. Gene expression profiling revealed cell line-dependent transcriptional responses, including modulation of components of the PI3K/AKT/mTOR pathway and EMT-associated markers. Collectively, our findings demonstrate that GO aerosol nanofilm alters PDAC cell morphology, viability, and migratory behavior in vitro. Surface-mediated modulation of tumor cell activity may represent a promising adjunct strategy for limiting residual cancer cell survival and metastatic potential.

## 1. Introduction

Pancreatic ductal adenocarcinoma (PDAC) is the most common type of pancreatic cancer and also has the poorest prognosis [1]. It originates in the exocrine pancreas and, although its etiology is not fully understood, it is suggested that PDAC may develop as a result of various factors, such as chronic inflammation, diabetes, viral or bacterial infections, and exposure to various carcinogens [2,3]. PDAC is very difficult to treat because it only manifests symptoms in the late stages and is a highly malignant cancer [1].

Adhesion of cancer cells to the extracellular matrix (ECM) plays a key role PDAC, maintaining tumor structure and enabling invasion during epithelial–mesenchymal transition (EMT) [4]. EMT is a process in which epithelial cancer cells acquire a mesenchymal phenotype, gaining the ability to penetrate the ECM and spread to distant organs [5]. These cells lose basement membrane attachment and, through polarization, enter blood and lymphatic vessels, forming metastatic foci [5].

Although EMT is essential in embryogenesis and tissue regeneration, it is tightly regulated during carcinogenesis [6]. It is controlled by multiple signaling pathways and genes such as *N-cadherin* and *E-cadherin*. *N-cadherin* is a marker of EMT and is associated with cancer progression, while reduced *E-cadherin* expression is typical of malignant tumors and may activate transcription factors regulated by the mTOR/PI3K/AKT pathway [7,8]. The mTOR/PI3K/AKT signaling pathway is a key regulator of EMT [9]. AKT kinase promotes this process and controls effectors like mTOR, responsible for cell growth and proliferation [10]. AKT is activated by PI3K, and its activity is inversely correlated with E-cadherin levels [11,12,13]. Increased AKT reduces E-cadherin expression, promoting EMT, while decreased AKT restores E-cadherin and limits cell migration [13]. Additionally, inflammatory cytokines, especially interleukins, also induce EMT. Chronic inflammation can create a pro-tumor environment, enhancing the secretion of cytokines such as interleukin-8 (IL-8), which promotes EMT, angiogenesis, and metastasis [14,15]. Given that EMT and associated signaling pathways enhance tumor progression and cellular proliferation, markers of proliferative activity become critical indicators of malignancy. An important marker of tumor malignancy is proliferating cell nuclear antigen (PCNA), which is involved in DNA replication, repair, and stability. Elevated PCNA expression supports rapid cancer cell proliferation, making it a key biomarker of aggressive tumors [16,17].

Given the characteristics described above, targeting factors that disrupt the signaling pathways associated with adhesion—even before a tumor forms—as well as those directly involved in migration and metastasis, could help inhibit the cells’ ability to invade. Nanomaterials that offer such effects include, amongst others, carbon allotropes such as graphene oxide (GO) [18,19]. GO is one of the allotropic forms of carbon [20]. Due to its unique properties, it may be a promising candidate for cancer therapies. Because GO occurs in the form of flakes with negatively charged surfaces (due to its functional groups), it can interact with proteins on the surface of cell membranes [21]. Consequently, GO flakes can attach to cell surfaces, which affects adhesion [21,22,23,24].

Thanks to its strength and the single-atom thickness of its flakes, this nanomaterial is also extremely sharp and can mechanically damage cell membranes, contributing to cell death [25]. Furthermore, as GO interacts electrostatically and hydrophobically with the abnormal membrane potentials of cancer cells, this alters membrane permeability and leads to the formation of pores, directly causing cell death [21,26]. In addition to physical damage, GO disrupts numerous molecular pathways and modulates genes responsible for tumor development that have been altered during carcinogenesis, including the previously mentioned mTOR/PI3K/AKT pathway [27,28]. This applies both to genes responsible for cell adhesion and to the pathways themselves, whose role is the migration and growth of cancer cells, which may affect their ability to invade [27,28].

In most cases, however, GO is used in the form of hydrocolloids in in vitro studies. Although this is a highly efficient method of delivering this nanomaterial, it has certain limitations. In vitro cell cultures, the aqueous GO suspension is present in a single environment alongside the culture medium. Consequently, growth factors and fetal bovine serum (FBS) proteins present in the medium, which are essential for cell growth, attach to the surfaces of the negatively charged functional groups of the GO flakes via various bonds (hydrogen bonds, electrostatic interactions, Van der Waals forces, π–π interactions), forming a so-called protein corona (PC) [21,29]. This means that growth factors in the culture medium are less readily available to the cells themselves due to their lower concentration, resulting in a further decline in viability, in addition to the GO effect itself [29]. Similarly, it has also been demonstrated that GO influences the occurrence of erythrocyte haemolysis due to interactions with proteins present in the blood [30].

GO flakes can mechanically damage cancer cells, but this may distort results in in vitro model studies [25]. Consequently, we decided to modify the method of GO administration by introducing a new approach involving the use of GO in aerosol form, which will allow us to circumvent the limitations associated with this nanomaterial in its colloidal form. Because the GO is used in aerosol form, it is possible to form a graphene nanofilm on the bottom of culture dishes during in vitro cell culture. This nanofilm is composed of overlapping, corrugated GO flakes; consequently, the resulting surface is not a uniform layer, which reduces cell adhesion by disrupting mechanotransduction signals [31]. This approach also minimizes the problem of the protein corona, as the flakes forming the nanofilm do not interact to such a great extent with the proteins present in FBS; consequently, during laboratory studies, the results obtained are derived primarily from the properties of GO. Consequently, we also minimize the risk of mechanical damage during in vitro culture, due to the presence of flakes suspended in the medium. Such surface—based approaches may be further explored in vivo as biomaterial coatings designed to locally modulate tumor microenvironment interactions, potentially limiting residual cancer cell survival and metastatic spread, as GO-based materials have already been proposed as multifunctional platforms for cancer therapy and biomaterial engineering [16,17,20,21]. The aim of this study was therefore to assess the effect of GO in aerosol form on the morphological and functional state of PDAC cells in vitro, and the effect of this nanomaterial on the expression of genes involved in EMT.

## 2. Results

### 2.1. Surface Morphology of Graphene Oxide Aerosol

**Figure 1:**
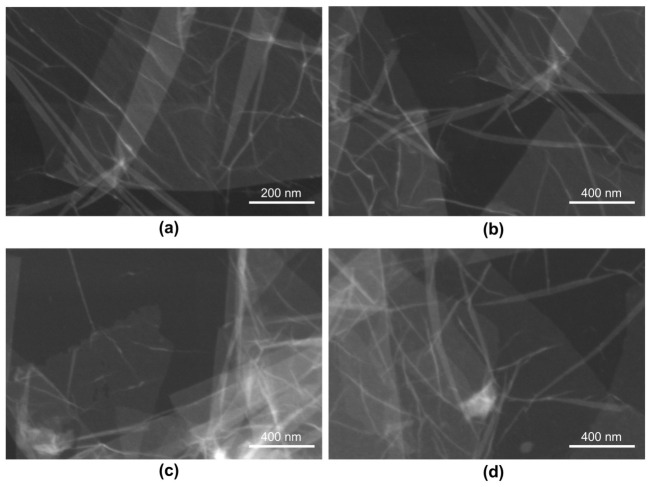
Ultrastructure of the surface of a graphene oxide aerosol with a concentration of 4.5 g/L. Images (**a**–**d**) show specimens imaged using STEM. Photographs at 200 nm (**a**) and 400 nm (**b**–**d**) scale.

Scanning transmission electron microscopy (STEM) analysis revealed that the GO aerosol consisted of thin, sheet-like nanostructures with irregular lateral dimensions and a characteristic wrinkled morphology (Figure 1a–d). The observed structures formed partially overlapping, semi-transparent lamellar flakes, indicative of exfoliated GO layers. At the nanoscale, the GO sheets exhibited a predominantly two-dimensional morphology with sharp, well-defined edges and variable lateral sizes extending to several hundred nanometers, as indicated by the 400 nm scale bar. The sheets appeared highly transparent in several regions and confirming their ultrathin structure. In contrast, darker areas corresponded to locally stacked or folded regions, indicating partial restacking of flakes following aerosol deposition. Wrinkling, bending, and edge folding were frequently observed, reflecting the intrinsic flexibility of GO sheets and the presence of structural defects introduced during oxidative exfoliation. The corrugated surface topology is consistent with the disruption of the sp^2^ carbon lattive and the incorporation of oxygen-containing functional groups, which induce lattice strain and increase interlayer spacing.

The observed ultrastrctural features are charasteristic of graphene oxide syntesized via modified Hummers’ method and are consistent with previously reported morphologies of chemically GO deridatives.

### 2.2. Zeta Potential

**Figure 2:**
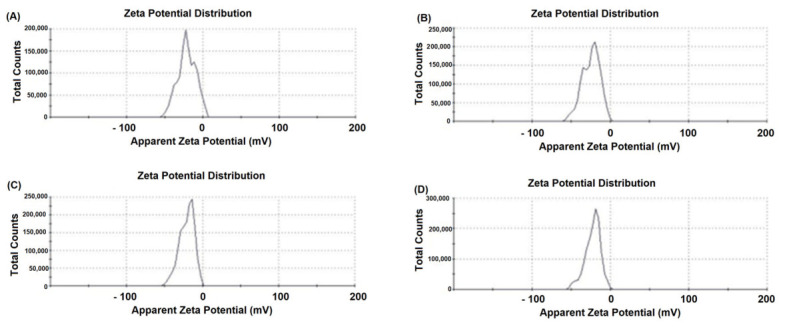
The zeta potential analysis of graphene oxide aerosol suspension. Used concentration: 4.5 g/L. Obtained results: −21.1 mV (**A**), −24.7 mV (**B**), −20.9 mV (**C**), −22.30 mV (**D**). The measurements were conducted using Zetasizer Nano analyzer (Malvern, Worcestershire, UK) and Zetasizer Software program (v8.02). Panels (**A**–**D**) show representative results from four independent replicates.

The electrokinetic properties of the GO aerosol were evaluated by measuring the zeta potential distribution (Figure 2A–D). All analyzed samples exhibited a narrow, monomodal distribution of zeta potential values in the negative range, confirming the anionic character of the GO surface. The mean zeta potential values were consistently below 0 mV, with peak maxima centered approximately in the range of −20 to −40 mV (depending on the measurement replicate). The negative surface charge of graphene oxide is primarily attributed to the presence of oxygen-containing functional groups, particularly carboxyl (-COOH/-COO^−^), hydroxyl (-OH), and epoxy (C-O-C) moieties introduced during oxidative exfoliation. Under aqueous conditions, partial ionization of carboxyl groups forms negatively charged carboxylate species, which significantly contribute to the dispersion’s overall electrostatic stabilization. Importantly, zeta potential values exceeding 30 mV (absolute value) are generally considered indicative of good colloidal stability due to electrostatic repulsion between particles. The measured values suggest that the GO aerosol exhibits moderate to high electrostatic stability, minimizing aggregation tendencies in aqueous suspension. The narrow distribution peaks further confirm the absence of distinct subpopulations with significantly different surface charges. Slight variations between panels (A–D) may reflect minor differences in dispersion conditions or measurement repeats; however, the overall electrokinetic behavior remained consistent across samples, demonstrating reproducibility of the GO aerosol preparation process. The strongly negative zeta potential is particularly relevant in the biological context, as surface charge influences protein adsorption, cellular uptake, membrane interactions, and overall biocompatibility. The anionic character of GO may modulate electrostatic interactions with positively charged domains of membrane proteins and extracellular matrix components, thereby affecting cell adhesion and signaling processes observed in subsequent in vitro experiments.

### 2.3. Dynamic Light Scattering Analysis

**Figure 3:**
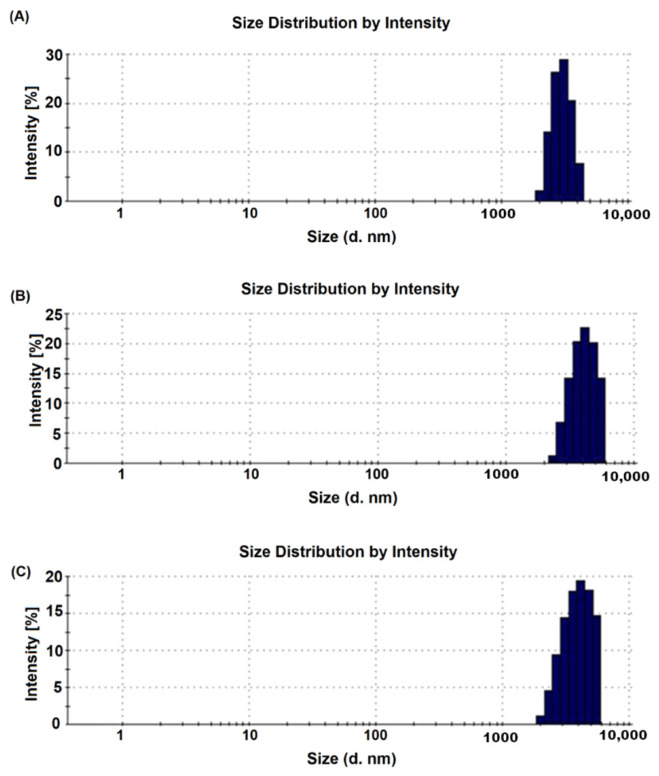
Results of DLS analysis of GO (**A**–**C**) aerosol. Panels (**A**–**C**) show representative results from three independent replicates.

The hydrodynamic size distribution of the GO aerosol was analyzed by dynamic light scattering (DLS) and is presented as an intensity-weighted distribution (Figure 3A–C). The measurement revealed a monomodal distribution with a dominant population of particles in the micrometer range. The main intensity peak was observed within the range of approximately 2–5 µm (2000–5000 nm), indicating that the aerosolized GO forms relatively large hydrodynamic structures in aqueous suspension. The narrow peak profile suggests moderate polydispersity and relatively uniform aggregate formation within the measured sample. The average PDI value for three independent measurements was 0.25 ± 0.094, indicating moderate polydispersity and a relatively narrow size distribution of the GO structures. Such values suggest good dispersion quality and colloidal stability of the nanomaterial, with a limited tendency toward aggregation. It is important to note that DLS reports the hydrodynamic diameter, which reflects not only the intrinsic lateral dimensions of individual GO sheets, but also their aggregation state and solvation shell in the dispersion medium. Given the two-dimensional morphology of GO, the measured values likely correspond to partially overlapped or stacked GO flakes rather than single-layer nanosheets. The absence of additional pronounced peaks in the submicron suggests limited fragmentation or formation of small nanoparticle fractions. Instead, the dominant contribution from larger hydrodynamic entities indicates a tendency toward sheet association in suspension, which may result from van der Waals interactions and hydrogen bonding between oxygen-containing functional groups.

### 2.4. Laser-Inducted Breakdown Spectroscopy Analysis

**Figure 4:**
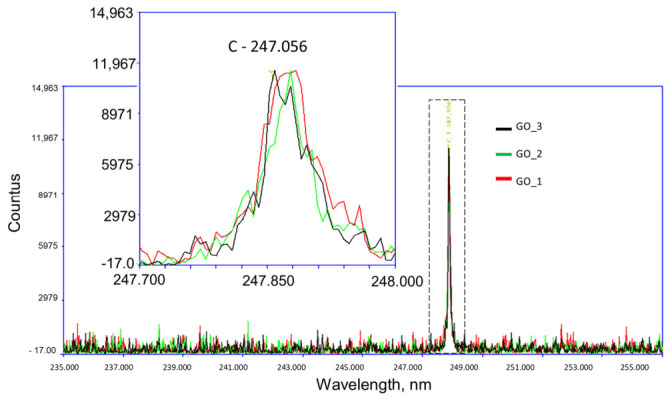
Results of LIBS analysis of GO aerosol, together with selected fragments of LIBS spectrum from carbon obtained in the 1st and 3rd laser pulses. The presented plots represent selected data derived from four independent measurements.

Figure 4 presents a representative fragment of the laser-induced breakdown spectroscopy (LIBS) spectrum recorded for the GO aerosol. The highlighted regions correspond to the characteristic emission lines of carbon at 247.056 nm, confirming the elemental composition of the analyzed material. Measurements were performed in three independent repetitions, and the results are presented by comparing the signal intensities obtained after the first and third laser pulses. The collected measurements show a relatively constant intensity of the carbon-derived pulse across all measurements.

### 2.5. Elemental Composition Analysis by EDX

**Figure 5:**
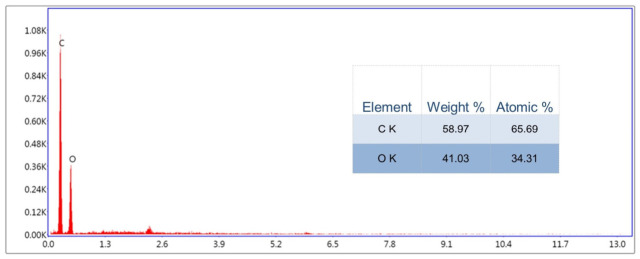
EDX spectrum and presentation of the average elemental composition value in the form of a graph. The measurements were conducted using scanning electron microscope (FEI Quanta 250 FEG; Thermo Fisher Scientific, Waltham, MA, USA) compressed with EDX detector. The presented spectrum corresponds to a representative result obtained from three independent experiments, with 10 spots analyzed per experiment.

The elemental composition of the GO aerosol was determined using energy-dispersive X-ray spectroscopy (EDX; DS-EDAX, LLC, Mahwah, NJ, USA) in conjunction with a scanning electron microscope (FEI Quanta 250 FEG; FEI, Hillsboro, OR, USA) (Figure 5). During the measurement, the average carbon content was 65.69% of the atomic composition, whilst oxygen accounted for 34.31%. In terms of mass percentage, the carbon-to-oxygen ratio was 58.97% to 41.03%. During analysis of the surface atomic composition, a relatively uniform distribution of these two elements was observed, as determined by their mass and atomic ratios. Figure 5 shows the presence of distinct peaks corresponding to carbon and oxygen, thereby confirming the carbon structure of GO enriched with oxygen-containing functional groups, which is consistent with its oxidized nature.

Furthermore, the predominance of carbon and oxygen, in the absence of detectable metallic impurities, confirms the material’s chemical purity and indicates that the oxidation of graphite during GO synthesis was successful.

### 2.6. FTIR-ATR and Raman Spectral Analysis of GO Aerosol

**Figure 6:**
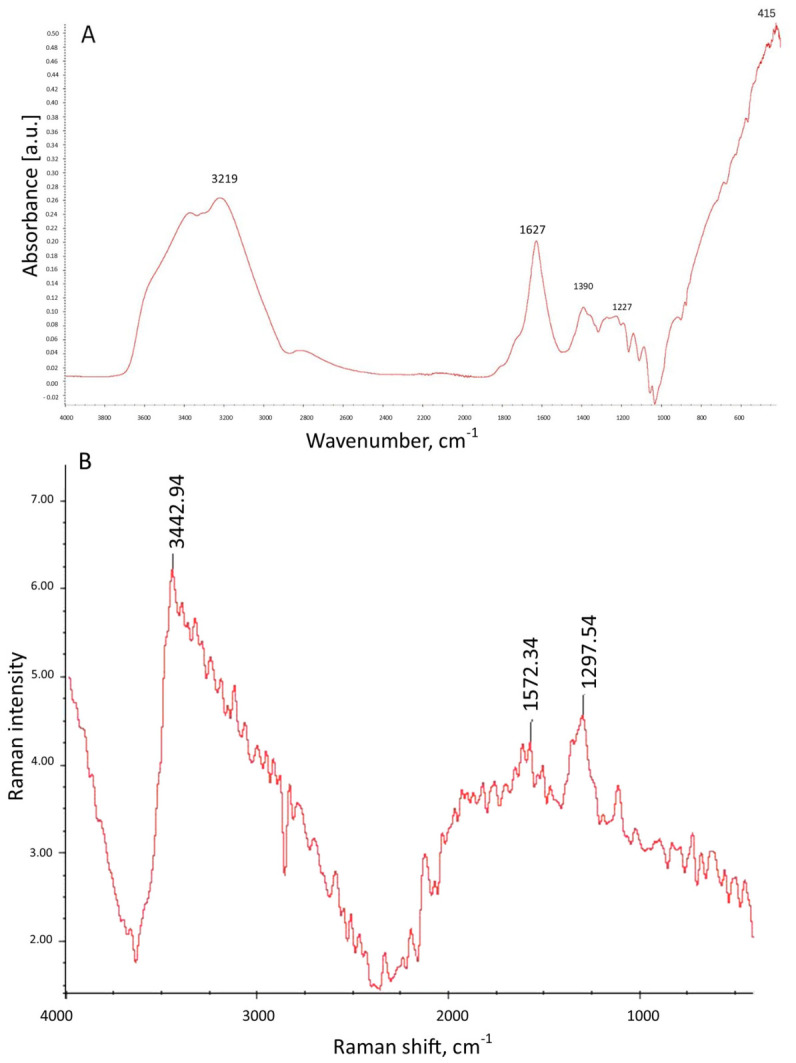
(**A**) ATR-FTIR spectrum of GO aerosol-derived nanofilm showing characteristic absorption bands at ~3219 cm^−1^ (O–H stretching), ~1627 cm^−1^ (C=C skeletal vibration and/or H–O–H bending), 1390–1086 cm^−1^ (C–O, C–O–C, and C–OH stretching vibrations), and ~415 cm^−1^. (**B**) Raman spectrum of graphene oxide showing the characteristic D band at ~1297.54 cm^−1^ and G band at ~1572.34 cm^−1^, corresponding to structural defects/disorder and sp^2^-hybridized carbon domains, respectively.

FTIR-ATR spectroscopy (Figure 6A) was performed to confirm the presence of oxygen-containing functional groups characteristic of GO. The recorded spectrum exhibited several distinct absorption bands corresponding to functional moieties introduced during the oxidative exfoliation of graphite. Complementary Raman spectroscopy (Figure 6B) further verified the structural features of GO, confirming the coexistence of disordered and graphitic domains. The Raman spectrum exhibited two characteristic bands typical for carbon-based nanomaterials: the D band (~1297.54 cm^−1^) and the G band (~1572.34 cm^−1^).

A broad intense band centered at approximately 3219 cm^−1^ was observed, which is attributed to the stretching vibrations of hydroxyl (-OH) groups. The broad nature of this band indicates extensive hydrogen-bonding interactions arising from both surface hydroxyl groups and adsorbed water molecules. This feature is a hallmark of oxidized graphene-based materials. A prominent band at 1627 cm^−1^ was assigned to the stretching vibrations of carbonyl (C=O) groups and/or skeletal vibrations of aromatic C=C domains. In GO, this region may also include contributions from bending vibrations of intercalated water molecules (δ O-H). The presence of this band confirms partial preservation of sp^2^-hybridized graphitic domains alongside oxygenated functionalities. The absorption band detected at 1390 cm^−1^ is attributed to deformation vibrations of hydroxyl groups (C-OH) and may also correspond to symmetric stretching of carboxylate (COO^−^) groups. This peak further supports the successful oxidation of the graphene structure. A characteristic band at 1227 cm^−1^ was assigned to C-O-C stretching vibrations of epoxy groups located on the basal plane of GO sheets. This band is considered one of the key spectral signatures confirming the formation of GO. Additional bands observed at 1139 cm^−1^ and 1086 cm^−1^ correspond to C-O stretching vibrations of alkoxy and alcohol functional groups. These peaks indicate the presence of oxygen-containing functionalities distributed across the GO surface. Finally, a low-wavenumber band at approximately 415 cm^−1^ can be attributed to skeletal deformation vibrations of the carbon framework, including out-of-plane bending modes of C-C bonds.

### 2.7. Morphology of Cell Lines

#### 2.7.1. Morphology of BxPC-3 Cell Line

**Figure 7:**
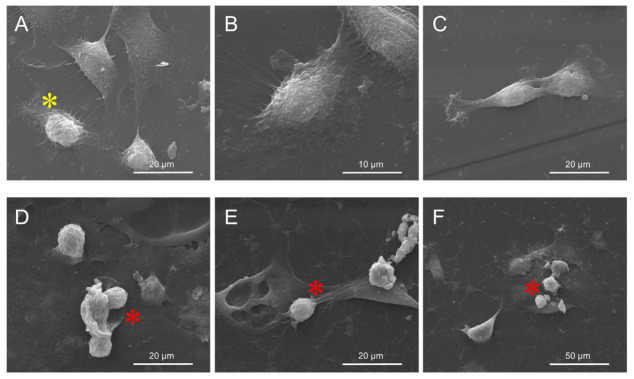
Cell morphology of BxPC-3 cells cultured on graphene oxide nanofilm (**D**–**F**) and control culture dishes without nanofilm (**A**–**C**). Red * indicates shortened cytoplasmic projections (**E**,**F**) and membrane disruption (**D**) in GO-exposed cells, and the yellow * indicates normal cellular morphology in control cells. Scale bars: 10–50 µm.

Scanning electron microscopy (SEM) analysis demonstrated that exposure to GO aerosol nanofilm markedly altered the morphology of BxPC-3 pancreatic ductal adenocarcinoma cells (Figure 7). In the control group (Figure 7A–C), cells exhibited a typical adherent epithelial-like morphology, characterized by a flattened shape, well-spread cytoplasm and numerous elongated cytoplasmic projections (filopodia and lamellipodia) mediating cell–substrate attachment. The plasma membrane appeared continuous and structurally intact. In contrast, BxPC-3 cells cultured on the GO nanofilm (Figure 7D–F) displayed pronounced morphological alterations. Cells exhibited clearly shortened and reduced cytoplasmic projections, accompanied by impaired spreading and partial loss of the typical polygonal morphology. Moreover, visible membrane irregularities and structural disruption were observed in several cells (Figure 7D), suggesting compromised membrane integrity. In some cases, cells appeared contracted and less firmly attached to the substrate. The observations indicate that the GO-modified surface disrupts cytoskeletal organization and cell–GO interactions, leading to structural destabilization of BxPC-3 cells.

#### 2.7.2. Morphology of AsPC-1 Cell Line

**Figure 8:**
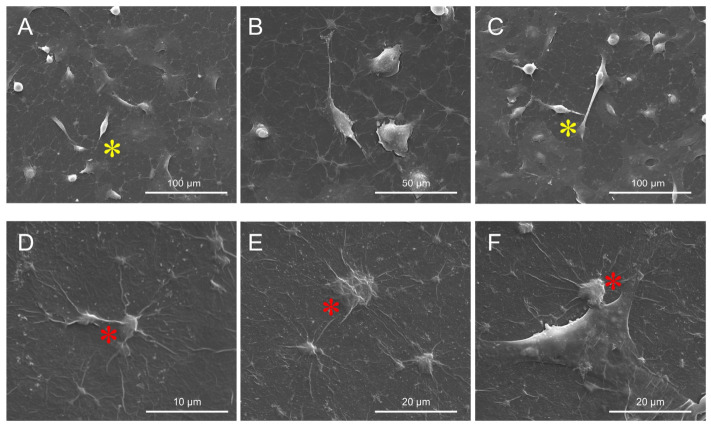
Cell morphology of AsPC-1 cells cultured on GO nanofilm (**D**–**F**) and control culture dishes without nanofilm (**A**–**C**). Red * indicates shortened cytoplasmic projections in GO-treated cells, and the yellow * indicates normal morphology in control cells. Scale bars: 10–100 µm.

SEM imaging of AsPC-1 cells similarly revealed morphological alterations following in vitro culture on GO aerosol nanofilm (Figure 8). Control AsPC-1 cells (Figure 8A–C) displayed an elongated and spindle-like morphology with well-developed cytoplasmic protrusions and extensive surface spreading, consistent with their highly migratory phenotype. Numerous thin filopodia were observed, facilitating cell adhesion and intercellular contacts. In contrast, cells cultured on the GO nanofilm (Figure 8D–F) demonstrated reduced cytoplasmic protrusions and a decreased spreading area. The cells appeared more compact and less elongated compared to controls. The shortening of filopodia and lamellipodia was particularly evident (Figure 8D–F), suggesting impaired cytoskeletal dynamics and altered adhesion properties. Collectively, these findings indicate that the GO aerosol nanofilm negatively affects the structural organization of both BxPC-3 and AsPC-1 pancreatic cancer cells, reducing protrusive activity and altering membrane morphology. Such changes may contribute to the decreased migratory potential observed in functional assays.

#### 2.7.3. Morphology of HFFF2 Cell Line

**Figure 9:**
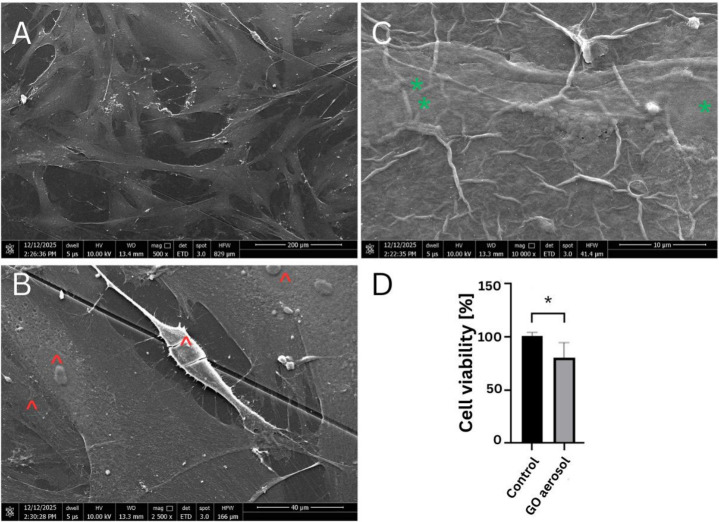
Cell morphology (**A***–***C**) and viability of HFFF2 (**D**) cells cultured on GO nanofilm (**C**,**D**) and control culture dishes without nanofilm (**A**,**B**). Red ^ indicates the nucleus of fibroblast cells; Green * indicates the nucleus in GO-treated cells. Scale bars: 40–200 µm. Statistically significant differences are indicated by asterisks (* *p* ≤ 0.05). Data represents mean ± SD of all results (n = 3).

SEM observation of HFFF2 morphology (Figure 9) showed that fibroblasts cultured under control conditions exhibited the characteristic elongated, spindle-shaped morphology that is typical of fibroblasts. Cells displayed numerous thin cytoplasmic protrusions and well-developed lamellipodia, indicative of strong substrate adhesion and preserved cytoskeletal organization. In contrast, fibroblasts cultured on the GO aerosol nanofilm were observed on a corrugated surface formed by deposited GO flakes. Although the cells retained their overall spindle-shaped morphology, a reduction in the length and number of cytoplasmic projections was evident. Lamellipodial structures appeared less pronounced compared with the control group. These morphological alterations suggest that the GO-modified substrate may partially affect fibroblast adhesion dynamics and cytoskeletal organization.

### 2.8. Viability of Pancreatic Cancer Cells and Fibroblasts

**Figure 10:**
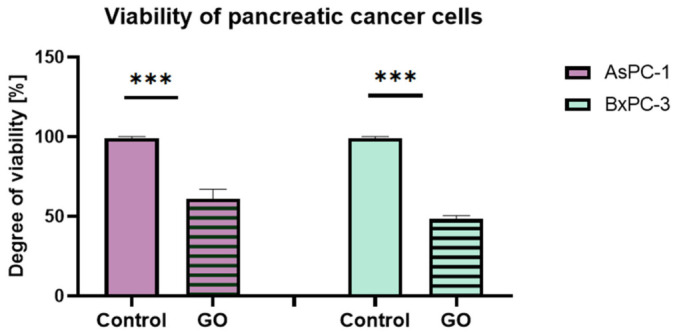
Cell viability results assessing the metabolic activity of AsPC-1 and BxPC-3 cells cultured on GO aerosol nanofilm relative to control groups. Statistically significant differences are indicated by asterisks (*** *p* ≤ 0.05). Data are presented as the mean ± standard deviation of all results (n = 3).

The metabolic activity of AsPC-1 and BxPC-3 PADC cell lines was evaluated using the XTT assay following exposure to the GO aerosol nanofilm (Figure 10). The results demonstrated that GO significantly reduced cell viability in both analyzed cell lines compared to their respective control groups cultured on unmodified culture dishes. In AsPC-1 cells, metabolic activity decreased by 42.87% relative to the control, indicating a substantial reduction in cell viability. An even more pronounced effect was observed in BxPC-3 cells, where viability decreased by 53.13% compared to untreated controls. The observed differences were highly statistically significant, confirming the cytotoxic or cytostatic effect of the GO-modified surface on pancreatic cancer cells. Notably, BxPC-3 cells were more sensitive to GO exposure than AsPC-1 cells, suggesting potential cell-line-dependent differences in susceptibility to surface-mediated nanomaterial interactions. The metabolic activity of HFFF2 fibroblasts was also evaluated using the XTT assay (Figure 9). The obtained results demonstrated a significant impact of both materials on fibroblast viability compared with the control group cultured on unmodified culture plates. Exposure to GO aerosol nanofilm resulted in a 20.39% reduction in cell viability relative to control conditions.

The metabolic activity of HFFF2 fibroblasts was evaluated using the XTT assay to determine the effect of the GO aerosol nanofilm (Figure 9). The obtained results demonstrated a significant impact of both materials on fibroblast viability compared with the control group cultured on unmodified culture plates. Exposure to GO aerosol nanofilm resulted in a 20.39% reduction in cell viability relative to control conditions.

### 2.9. Migration of Pancreatic Cancer Cells

**Figure 11:**
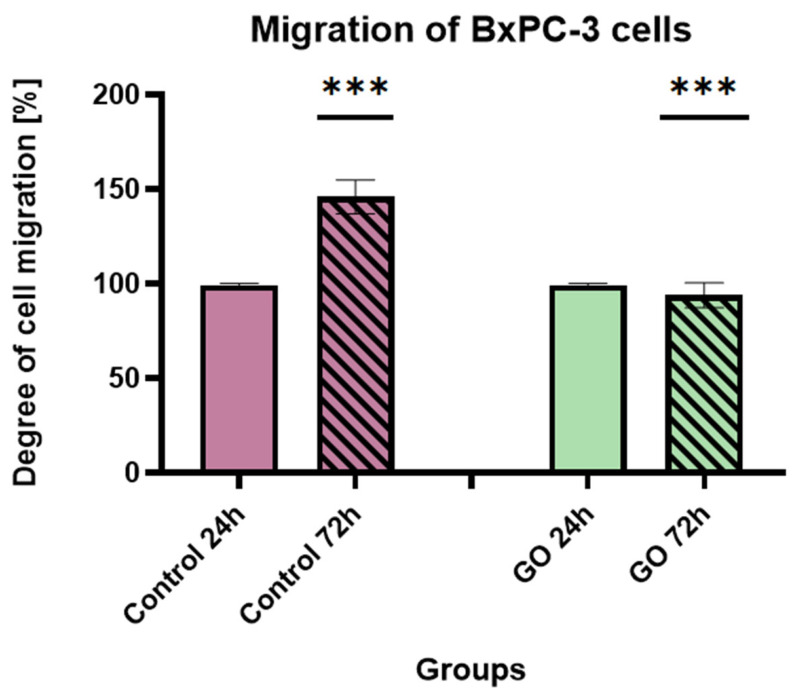
Degree of migration of BxPC-3 cells after 24 and 72 h in control conditions and on GO nanofilm. Statistically significant differences are indicated by asterisks (*** *p* ≤ 0.05). Data are presented as the mean ± standard deviation of all results (n = 3).

**Figure 12:**
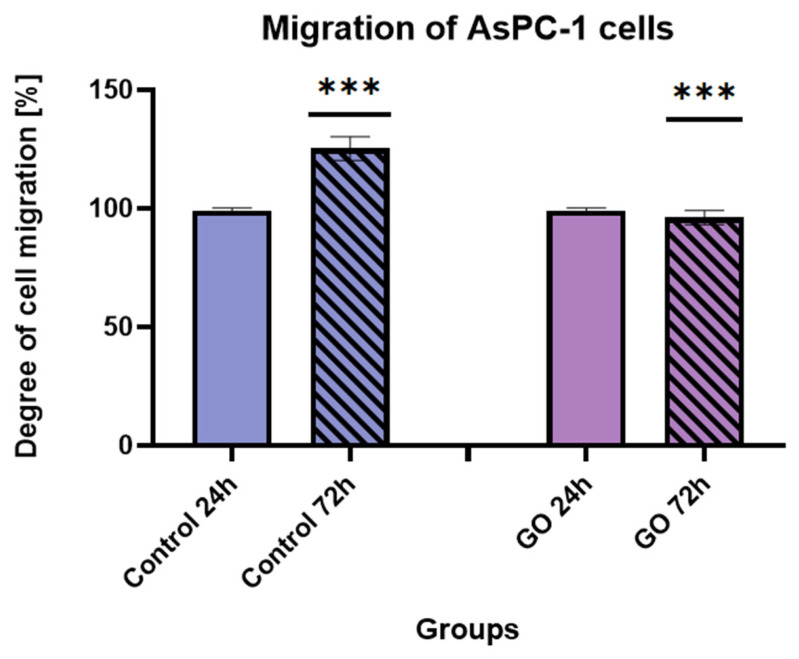
Degree of migration of AsPC-1 cells after 24 and 72 h in control conditions and on GO aerosol nanofilm. Statistically significant differences are indicated by asterisks (*** *p* ≤ 0.05). Data are presented as the mean ± standard deviation of all results (n = 3).

The migratory potential of pancreatic ductal adenocarcinoma cells cultured on a GO aerosol nanofilm was evaluated using a wound-healing assay over 72 h (Figure 11 and Figure 12). Cells cultured on GO-modified surfaces exhibited reduced adhesion and spreading compared to controls. After 72 h, partial cell detachment from the GO nanofilm was observed, resulting in a reduced number of adherent cells in the wound area. In contrast, control groups cultured on unmodified surfaces demonstrated progressive wound closure and increased cell coverage over time. In the BxPC-3 cell line, a clear divergence between control and GO-treated groups was observed (Figure 11). In the control group, cell migration increased by 37.21% after 72 h compared to the 24-h time point, indicating active wound closure and preserved migratory capacity. In contrast, cells cultured on the GO nanofilm exhibited a 13.5% decrease in migration after 72 h relative to 24 h. This reduction reflects impaired cell spreading and diminished ability to repopulate the wound area. The observed results showed that GO surface functionalization markedly suppresses the migratory potential of BxPC-3 cells.

A similar, although less pronounced, trend was observed in the AsPC-1 cell line (Figure 12). In the control group, migration increased by 20.16% from 24 h to 72 h, reflecting progressive wound closure. In contrast, AsPC-1 cells cultured on the GO nanofilm exhibited a 6.91% decrease in migration over the same time period. The images showing the surface morphology are provided in the Supplementary Material (Figure S1). This reduction suggests that GO exposure interferes with cytoskeletal dynamics and cell–GO interactions, thereby limiting migratory behavior.

### 2.10. Gene Expression Analysis in Pancreatic Cancer Cells

#### 2.10.1. Gene Expression Analysis in BxPC-3 Cells

**Figure 13:**
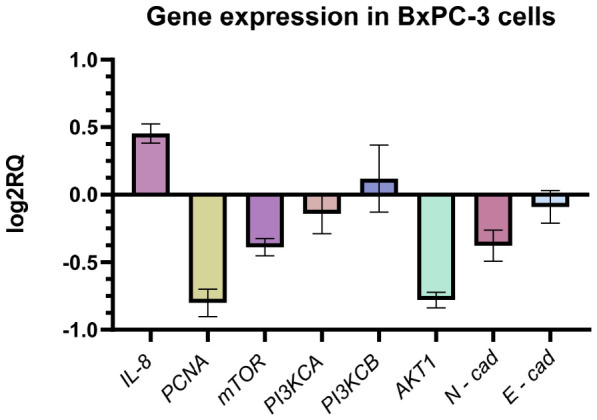
Relative gene expression in BxPC-3 pancreatic adenocarcinoma cells cultured on GO nanofilm compared to control cells. Expression levels were calculated using the 2^−ΔΔCt^ method and normalized to GAPDH. No statistically significant differences were detected (*p* ≥ 0.05). Data are presented as mean ± SD (n = 3) of log2-transformed relative expression (log2RQ).

Relative gene expression analysis in BxPC-3 cells cultured on GO aerosol nanofilm revealed distinct transcriptional modulation compared to control conditions (Figure 13). Expression levels were normalized to *GAPDH* and calculated using the 2^−ΔΔCt^ method, with results presented as log_2_RQ values. As shown in Figure 11, GO exposure was associated with a marked downregulation of several proliferation- and EMT-related genes, including *PCNA*, *mTOR*, *PI3KCA*, *AKT1*, and *N-cadherin*. The strongest decrease was observed for *PCNA* and *AKT1,* suggesting suppression of proliferative signaling and attenuation of the PI3K/AKT pathway. Additionally, reduced N-cadherin expression may indicate partial inhibition of mesenchymal features associated with epithelial–mesenchymal transition (EMT). Interestingly, a modest upregulation of *IL-8* was detected, whereas *PI3KCB* expression showed a slight increase. The expression of *E-cadherin* remained close to baseline, indicating no strong reactivation of epithelial characteristics. The observed transcriptional profile suggests a trend toward reduced proliferative and migratory signaling in BxPC-3 cells following GO exposure. These molecular trends are consistent with the decreased metabolic activity and impaired migration observed in functional assays.

#### 2.10.2. Gene Expression Analysis in AsPC-1 Cells

**Figure 14:**
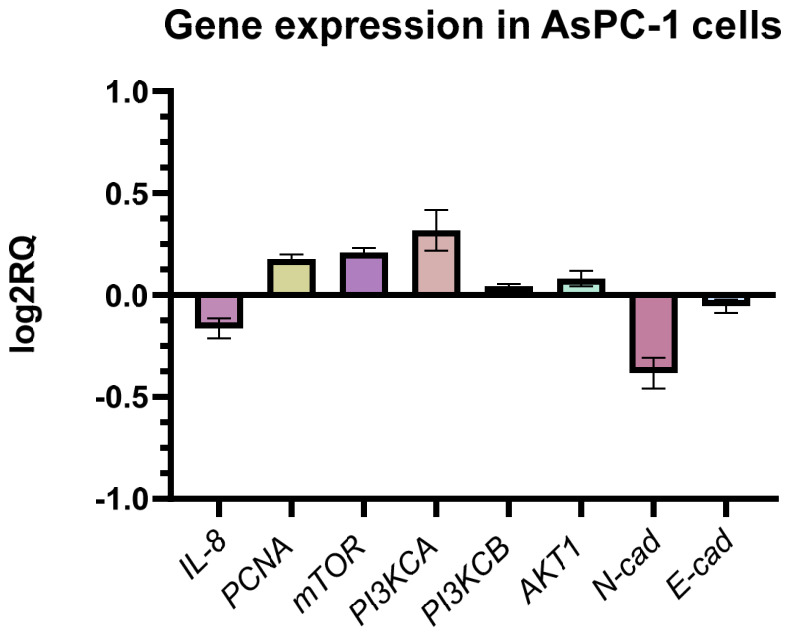
Relative gene expression in AsPC-1 pancreatic adenocarcinoma cells cultured on GO nanofilm compared to control cells. Expression levels were calculated using the 2^−ΔΔCt^ method and normalized to GAPDH. No statistically significant differences were observed (*p* ≥ 0.05). Data are presented as mean ± SD (n = 3) of log2-transformed relative expression (log2RQ).

In contrast to BxPC-3 cells, AsPC-1 cells exhibited a different transcriptional response to GO aerosol nanofilm exposure (Figure 14). The data show a general upward trend in the expression of several genes involved in proliferative and EMT-associated pathways, including PCNA, mTOR, and PI3KCA. Moderate increases were also observed for AKT1. These changes may reflect activation of survival or compensatory signaling mechanisms in response to GO-induced stress. Notably, N-cadherin expression was reduced, while E-cadherin levels remained relatively unchanged. The decrease in N-cadherin suggests that, despite partial activation of proliferative pathways, mesenchymal traits may not be strongly enhanced. Additionally, IL-8 expression was slightly reduced compared to controls. Similarly to BxPC-3 cells. However, the opposing directional trends between the two cell lines indicate cell line-dependent molecular responses to GO-modified substrates.

#### 2.10.3. Gene Expression Analysis in HFFF2

**Figure 15:**
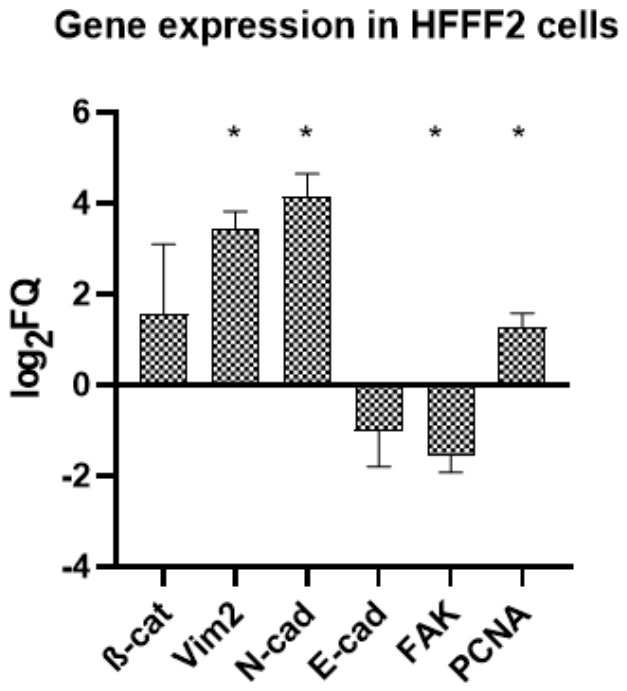
Relative mRNA expression levels of adhesion- and proliferation-related genes in HFFF2 fibroblasts cultured on GO aerosol-derived nanofilm compared with control conditions. Expression levels of *β-catenin* (*β-cat*), *vimentin* (*Vim2*), *N-cadherin* (*N-cad*), *E-cadherin* (*E-cad*), *focal adhesion kinase* (*FAK*), and PCNA are presented as log_2_ fold change (log_2_FQ) values calculated using the ^ΔΔ^Ct method. Statistically significant differences are indicated by asterisks (* *p* ≤ 0.05). Data are presented as mean ± SD (n = 3) of log2-transformed relative expression (log2RQ).

Gene expression analysis of HFFF2 fibroblasts cultured on GO aerosol nanofilm (Figure 15) demonstrated coordinated modulation of genes associated with both proliferation and cell adhesion compared with control conditions. The results, expressed as log2 fold change (log2RQ), revealed increased expression of the proliferation marker *PCNA*, indicating preserved proliferative capacity and maintained cell cycle activity in fibroblasts exposed to GO. In parallel, adhesion-related markers *N* and *E-cadherin*, and *focal adhesion kinase* (*FAK*)- showed elevated transcript levels relative to the control group. The most pronounced upregulation was observed for N-cadherin, suggesting enhanced intercellular interaction potential and possible adaptive strengthening of adhesion-related signaling pathways in response to the GO-modified substrate. In contrast, *E-cadherin* expression was slightly reduced compared with control conditions.

## 3. Discussion

In this study, we demonstrate that GO applied in aerosol form, generating a substrate-bound nanofilm, can significantly influence the morphological and functional behavior of pancreatic ductal adenocarcinoma cells, suggesting a microenvironment-focused mechanism of action distinct from classical nanoparticle exposure models. The ultrastructural features observed in the present study—wrinkled, multilamellar, partially overlapping GO flakes- are consistent with the canonical morphology of GO synthesized via modified Hummers’ method.

Similar topographical characteristics have been widely described in suspension-derived GO systems [30,31]. However, an important distinguishing feature of the present work is that GO was applied in aerosol form, resulting in the formation of a substrate-bound nanofilm rather than a colloidal dispersion interacting with cells in suspension. In classical colloidal systems, GO flakes remain dispersed in aqueous media and interact dynamically with cell membranes, often inducing oxidative stress, membrane penetration, or internalization [32]. In contrast, aerosol-deposited GO forms a relatively stable, spatially fixed, multilayered coating that primarily modulates cell behavior through surface topography, mechanical heterogeneity, and adhesion-dependent signaling, rather than through particle internalization. This distinction is critical for interpreting biological responses, as substrate-bound GO predominantly exerts mechanobiological effects, while colloidal GO frequently induces chemically mediated cytotoxicity. The hydrodynamic size (~3163 nm) determined by DLS in the present study reflects stacked or associated GO lamellae in dispersion prior to deposition. Such micron-scale effective diameters are consistent with reports showing that 2D nanosheets tend to form aggregates in aqueous environments due to van der Waals interactions and hydrogen bonding [33]. Importantly, once aerosolized and deposited, these associated sheets reorganize into a discontinuous but macroscopically uniform film. This behavior differs substantially from dilute colloidal systems, where individual flakes remain more dispersed and mobile. Similarly, the moderately negative zeta potential observed in this study falls within the range typically reported for aqueous GO suspensions (−20 to −50 mV) [34]. In colloidal systems, this surface charge mainly governs electrostatic stability and interaction with serum proteins. In contrast, in aerosol-derived nanofilms, the surface charge contributes more directly to protein adsorption patterns and integrin-mediated adhesion at the solid–liquid interface [35]. The LIBS and EDX analyses further support the structural integrity and compositional consistency of the deposited film. Depth-dependent variations in oxygen and carbon signal intensity observed in LIBS measurements align with previous reports describing oxygen-enriched surface layers in GO coatings [36]. Such stratification is particularly relevant in aerosol systems, where rapid solvent evaporation during spraying can promote surface enrichment of oxygen-containing functional groups. In contrast, colloidal GO dispersions tend to exhibit more homogeneous chemical exposure across suspended flakes. Taken together, the physicochemical profile of the GO aerosol nanofilm differs functionally from classical suspension-based GO models. While both systems share core structural characteristics of GO sheets, aerosol deposition creates a mechanically active, surface-bound nanostructured interface that more closely resembles engineered extracellular matrix modifications than nanoparticle exposure scenarios. This distinction is particularly relevant in PDAC cancer, where cell adhesion, matrix stiffness sensing, and integrin-driven PI3K/AKT/mTOR signaling critically regulate survival and epithelial–mesenchymal transition [37,38].

The biological responses observed in the present study should be interpreted within the broader framework of GO-mediated modulation of cancer cell behavior, accounting for both the physicochemical presentation and cell-specific characteristics. Unlike most published investigations that evaluate GO in suspension, the current work employed an aerosol-derived, substrate-bound nanofilm, which fundamentally alters the mode of cellular interaction. This distinction is critical because surface-immobilized GO predominantly influences adhesion-dependent signaling and mechanotransduction rather than inducing classical nanoparticle-mediated oxidative stress or intracellular accumulation mechanisms described in suspension systems [23]. The reduction of cytoplasmic protrusions observed in both BxPC-3 and AsPC-1 cells is consistent with previous reports demonstrating that nanoscale surface roughness and discontinuous topography impair focal adhesion maturation and cytoskeletal organization [39]. Filopodia and lamellipodia are essential for integrin clustering, mechanosensing, and directed migration; therefore, their attenuation suggests disrupted cell–substrate anchoring [40]. In BxPC-3 cells, the additional observation of membrane degradation indicates heightened structural stress, potentially reflecting differential membrane composition or cytoskeletal tension between the two lines. Comparable morphology-dependent responses were reported by Wójcik et al. (2021) [41], who described concentration-dependent structural alterations under GO exposure. However, the significantly higher GO concentration used in the present study may explain the more pronounced ultrastructural effects. The marked reduction in metabolic activity observed in both PDAC cell lines exceeds the decreases in viability reported in several suspension-GO-based studies. For example, Wójcik et al. (2021) [41] observed viability levels remaining above 80% at lower GO concentrations, while Nasiłowska et al. (2020) [29] reported only minimal viability reduction on breast cancer cells cultured on GO nanofilm. These discrepancies highlight the importance of exposure format, concentration, and intrinsic cellular phenotype. In PDAC, survival signaling is tightly linked to integrin-mediated activation of the PI3K/AKT/mTOR pathway. Disruption of focal adhesion complexes can initiate anoikis-like responses and suppress proliferative signaling [42]. Therefore, the viability reduction observed in the present study is likely driven not solely by chemical cytotoxicity but by impaired mechanotransduction resulting from the nanostructured surface. The inhibition of migration further supports this mechanobiological interpretation. Migration in PDAC is strongly influenced by substrate stiffness, adhesion strength, and integrin-dependent signaling networks [43]. The mechanically heterogeneous GO nanofilm likely interferes with traction force generation and coordinated cytoskeletal remodeling required for wound closure. The more pronounced migration suppression in BxPC-3 compared to AsPC-1 may reflect inherent differences in aggressiveness and EMT status between the cell lines, suggesting that baseline molecular programming modulates sensitivity to topographical perturbation. The divergent transcriptional trends observed between BxPC-3 and AsPC-1 cells further emphasize the cell line-dependent nature of these responses. In BxPC-3 cells, the downward trend in mTOR/PI3K/AKT pathway components aligns with reduced proliferative capacity and is consistent with the literature, which demonstrates that suppression of this axis inhibits PDAC progression [44]. The concomitant decrease in *PCNA* expression supports reduced replication potential. Increased IL-8 expression may indicate a stress-associated inflammatory response, which, if sustained, could contribute to diminished cellular fitness. In contrast, AsPC-1 cells tended to maintain or increase expression of components of the proliferative pathway despite functional impairment. Such behavior may represent compensatory activation of survival signaling. Zheng et al. (2023) [45] demonstrated that low GO concentrations can activate the PI3K/AKT/mTOR cascade via integrin αV, suggesting that GO-induced signaling is highly context dependent and influenced by local concentration, integrin repertoire, and presentation format. In a substrate-bound configuration, effective cellular exposure may differ substantially from suspension models, potentially explaining the complex transcriptional patterns observed. The consistent reduction in cadherin expression across both cell lines indicates disrupted adhesion dynamics and may reflect secondary modulation of EMT-associated pathways. Mechanical perturbation of cell–substrate interactions is increasingly recognized as a regulator of epithelial–mesenchymal transition [46]. Thus, the molecular trends identified here likely represent downstream consequences of altered adhesion and mechanosensing rather than direct genotoxic effects. To provide additional context for the cancer-specific responses, the impact of GO aerosol nanofilm was also assessed in non-malignant human fibroblasts (HFFF2), which serve as a physiological reference model for stromal cells. Unlike the significant functional suppression noted in PDAC cells, fibroblasts demonstrated a more moderate reaction to GO exposure. Although a reduction in metabolic activity was detected, fibroblasts largely preserved their typical morphology and maintained proliferative activity, as indicated by increased expression of *PCNA* and *Ki-67*. Interestingly, adhesion-related genes, including *β-catenin*, *vimentin*, *N-cadherin*, and *FAK,* were upregulated in HFFF2 cells cultured on GO nanofilms, suggesting that these cells were adapting to GO nanofilm interactions rather than undergoing adhesion. These findings suggest that GO nanofilm may exert differential, cell-type-dependent effects, impairing adhesion-dependent survival mechanisms in PDAC cells while allowing adaptive remodeling responses in non-malignant fibroblasts. Such selectivity is particularly relevant in the context of tumor microenvironment modulation, as stromal fibroblasts are key regulators of extracellular matrix organization and mechanical signaling in pancreatic cancer. The preserved proliferative and adhesion-related gene activity in fibroblasts may indicate that GO nanofilm does not induce generalized cytotoxicity but rather interferes preferentially with cancer-associated mechanotransductive pathways. The consistent directional trends, combined with pronounced functional changes, strongly suggest that GO aerosol nanofilm modulates PDAC cell behavior primarily through surface-mediated mechanotransductive mechanisms. This interpretation aligns with the broader understanding that the tumor microenvironment’s physical properties critically regulate cancer progression [41].

## 4. Materials and Methods

### 4.1. Physicochemistry of Graphene Oxide Aerosol (GO)

The GO aerosol with a concentration of 4.5 g/L was produced using the Bag-On-Valve (BOV) method from a commercially purchased GO suspension (G-Flake^®^, Łukasiewicz Research Network—Institute of Microelectronics and Photonics, Warsaw, Poland), which allows for the application of material in aerosol form [29]. The design consisted of a BOV Crimper aerosol valve connected to a four-layer foil bag. The bag included: polyethylene, aluminum, nylon, and polypropylene [29]. The entire system included an Under The Cup pre-aeration (UTC) section and a BOV valve that blocked the aluminum container. Thanks to the sealing of the entire system, it was possible to introduce air at 1–3 bar between the bag and the container. Then, the bag was filled with the GO suspension to achieve a pressure of 9 bars [29]. Prior to cell culture experiments, all samples were sterilized by gamma (γ) irradiation following standard sterilization protocols for biomaterials. The prepared GO aerosol was used for physicochemical research and in model in vitro studies on BxPC-3, AsPC-1, and HFFF2 cell lines.

### 4.2. Characterization of GO Aerosol

#### 4.2.1. Surface Morphology of Graphene Oxide Aerosol

Ultrastructural studies of GO aerosol were conducted using the STEM method (Scanning Transmission Electron Microscopy) with a FEI Quanta 250 FEG scanning electron microscope (FEI, Hillsboro, OR, USA). We used the GO aerosol with a specific model of centrifuge on a rotating plate at a speed of 2000 RPM (revolutions per minute) to remove any excess aerosol. After preparation, the sample was placed on the stage of an electron microscope and allowed to dry before analysis.

#### 4.2.2. Zeta Potential

The Zetasizer Nano analyzer (Malvern Panalytical, Malvern, Worcestershire, UK) and Zetasizer Software were used to measure the electrokinetic potential (Zeta potential). The analysis was performed in U-shaped cuvettes containing an aqueous suspension of graphene oxide aerosol. During the measurement, the sample is subjected to an electric field, in which the particles begin to move at their characteristic speed. The measurement was performed in three repetitions under constant measurement conditions.

#### 4.2.3. Dynamic Light Scattering Analysis

Dynamic Light Scattering (DLS) measurements were also performed using a Zetasizer Nano analyzer (Malvern Panalytical, Malvern, Worcestershire, UK) and Zetasizer Software. This method is used to assess the size of particles suspended in a liquid. The principle of operation is based on illuminating the suspended particles with laser beams, which causes the light to scatter and the signal to be collected by a detector. The speed of nanoparticle movement is measured by particle vibrations, known as Brownian motion. The measurement is then converted into a size distribution. The signal obtained is altered by the Brownian motion of the nanoparticles. The measurement was performed in three repetitions under constant conditions.

#### 4.2.4. LIBS Analysis

A Quantel Nd:YAG pulsed laser (model Brio; Bozeman, MT, USA) was used to generate the plasma. The wavelength of the radiation used for the experiments was 1064 nm. These radiation-generated pulses have a duration of 4 ns. To enable time-resolved measurements, the plasma-generated radiation was detected by an ESA 4000 echelle spectrometer (LLA Instruments GmbH & Co. KG, Berlin, Germany), which recorded spectra over 20 ns to 16 ms at 200–800 nm. Detection was possible thanks to the Kodak KAF 1001 CCD matrix with an ICCD amplifier (Eastman Kodak Company, Rochester, NY, USA), which was equipped with the ESA 4000 device (EnviroESCA Ltd., Dublin, Ireland). The spectral resolution of the entire detection system was λ/Δλ~20,000 [31]. To perform the measurement, a thin layer of GO aerosol at 4.5 g/L was applied to a microscope slide, which was then dried in a vacuum dryer (Vacucell, MMM Group, Planegg, Germany) at 60 °C for approximately 15 min. The prepared sample was placed in the analysis device and the measurement was performed. Each measurement was performed in four independent replicates, and the presented plots show representative data derived from these measurements.

#### 4.2.5. EDX Analysis

Energy-dispersive X-ray spectroscopy (EDX/EDS) was used to analyze the elemental composition of GO aerosol, using an EDX detector with a Quanta 250 FEG FEI microscope (Hillsboro, OR, USA). Parameters used during the EDX analysis: spot 4.5, 30 kV [47]. The analysis was carried out in three repetitions. There were 10 spots analyzed in each repetition.

#### 4.2.6. Fourier Transform Infrared (FTIR) Spectroscopy

The FTIR spectra of dried samples were recorded using an FTIR spectroscope with a diamond ATR pickup (Nicolet 6700, Thermo Scientific). All samples were prepared by a single application of the GO aerosol on a microscope slide and drying at 40 °C. Each spectrum was ratioed to a background spectrum previously registered with an empty measuring chamber. This was done to remove the effect of carbon dioxide and water vapor present in the laboratory air. Each spectrum was recorded in three independent replicates.

#### 4.2.7. Raman Spectroscopy

Raman spectroscopy measurements were performed using a Nicolet iS50 spectrometer (Thermo Fisher Scientific) equipped with an InGaAs detector and a CaF_2_ beam splitter. The spectra were recorded in the range of 400–4000 cm^−1^ with a spectral resolution of 4 cm^−1^. A total of 200 scans were collected for each sample, with an acquisition time of 418.6 s. The Raman laser frequency corresponded to 9392.72 cm^−1^. The collected spectra were subjected to automatic baseline correction (polynomial order 2, 20 iterations) and smoothing procedures to reduce noise and improve signal clarity. All measurements were performed in three independent replicates. The obtained spectra were analyzed in terms of characteristic graphene oxide bands.

### 4.3. In Vitro Studies on the Cell Lines

#### 4.3.1. In Vitro Culture

Cell cultures of the BxPC-3 (CRL-1687) and AsPC-1 (CRL-1682) lines obtained from the American Type Culture Collection were maintained in 75 cm^3^ culture bottles. The culture medium used was sterile RPMI 1640 (R8758, Sigma Aldrich, St. Louis, MO, USA) containing L-glutamine and sodium bicarbonate with 10% FBS (Fetal Bovine Serum, Sigma Aldrich, St. Louis, MO, USA) and 1% antibiotic: streptomycin and penicillin (P4333, Sigma Aldrich, St. Louis, MO, USA).

The human fetal foreskin fibroblast cell line HFFF2 (catalog no. 86031405) was obtained from Sigma-Aldrich (St. Louis, MO, USA). HFFF2 cells represent a non-transformed human fibroblast model commonly used in studies of cell biology, toxicology, tissue engineering, and biomaterial biocompatibility assessment. HFFF2 fibroblasts display a characteristic spindle-shaped morphology with elongated cytoplasmic extensions supporting strong surface adhesion. These cells exhibit high proliferative capacity and responsiveness to environmental stimuli. In the present study, HFFF2 cells were used as a non-malignant reference control to evaluate whether the GO aerosol-derived nanofilm exerts selective effects on cancer cells compared with normal human fibroblasts.

#### 4.3.2. Preparation of the Surface for Cell Cultivation

6- 12-, and 96-well culture plates were used to conduct the tests. GO aerosol at a concentration of 4.5 g/L was applied to the bottom surface of the culture vessels to form a nanofilm, and then left to dry under sterile conditions in a laminar flow cabinet (BSC II ESCO Airstream (ESCO)) for 12–16 h. All culture vessels were prepared in four independent replicates, and only those with uniform and consistent GO nanofilm coverage were selected for further experiments. To initiate cancer cell cultures on 6-, 12-, and 96-well culture dishes, the cells needed to be detached from the surface of the culture bottles. This was achieved by adding 2 mL of a 0.25% trypsin solution containing EDTA (Sigma Aldrich, St. Louis, MO, USA) after removing the culture medium and rinsing with PBS (Phosphate-Buffered Saline, Sigma Aldrich, St. Louis, MO, USA). Following the addition of trypsin, the culture bottles were incubated for 5–10 min in a cell culture incubator set at 5% CO_2_ and 37 °C (Memmert GmbH + Co. KG, Schwabach, Germany). Once the cells were separated from the bottom of the culture vessels, trypsin’s enzymatic activity was neutralized by adding 2 mL of culture medium supplemented with fetal bovine serum (FBS) and antibiotics. Next, the cells were seeded into the appropriate culture vessels with GO aerosol nanofilms at concentrations ranging from 5 × 10^3^ to 5 × 10^5^. The cultures were maintained in 6-, 12-, and 96-well plates under standard conditions until ready for analysis.

#### 4.3.3. Morphological Analysis of Pancreatic Cancer Cells

The morphology of cancer cells was assessed using scanning electron microscopy (SEM). Cell cultures grown on 6-well plates were used for analysis. Cells were seeded at a density of 1 × 10^4^/well and incubated in a cell culture incubator (Memmert, Schwabach, Germany) under standard conditions (5% CO_2_ and 37 °C). After 24 h of incubation, the cells were fixed with 2.5% glutaraldehyde (Sigma, St. Louis, MO, USA) for 30 min at room temperature. The cell cultures were then rinsed with phosphate buffer (Sigma, St. Louis, MO, USA) and contrasted with osmium tetroxide (OsO_4_, Sigma Aldrich, St. Louis, MO, USA) for 2 h at room temperature. To remove the osmium tetroxide, the cells were rinsed with phosphate buffer (Sigma, USA). They were then dehydrated in increasing concentrations of ethanol (50% to 99.98%) and dried by critical-point drying (Polaron CPD 7501, Quorum Technologies, Newhaven, East Sussex, UK). For further preparation, the sample was sputter-coated with gold ions in a vacuum sputter coater (JEE-4C, JEOL Ltd., Tokyo, Japan). The prepared sample was imaged in a scanning electron microscope at 1 kV (FEI Quanta 200). All measurements were conducted using 10 independent replicates.

#### 4.3.4. Viability Analysis on Pancreatic Cancer Cells

The XTT metabolic activity test (XTT Cell Viability Assay Kit, Biotum, San Francisco, CA, USA) was used to assess the viability of cancer cells. Cell cultures grown on 96-well plates were used to analyze viability. Cells seeded at a density of 5 × 10^3^/well were incubated for 24 h in a cell culture incubator (5% CO_2_, 37 °C). Then, under sterile conditions, 25 µL of tetrazolium salt solution was added to the wells of the culture vessels. After another 2 h of incubation, the cells were analyzed spectrophotometrically using an Infinite M200 plate reader (Tecan, Männedorf, Switzerland) at 450–500 nm. Cell viability was expressed as a percentage (ODtest—ODblank)/(ODcontrol—ODblank), where “ODtest” is the OD of cells exposed to GO aerosol, “ODcontrol” is the OD of the control sample, and “ODblank” is the OD of wells without GO. All measurements were performed in triplicate.

#### 4.3.5. Migration Analysis of Pancreatic Cancer Cells

The migratory capacity of human pancreatic cancer cell lines BxPC-3 and AsPC-1 in response to GO aerosol nanofilm exposure was evaluated using a wound healing assay. All measurements were performed in three technical replicates.

##### Cell Preparation

Cells were cultured under standard conditions (37 °C, humidified atmosphere containing 5% CO_2_) in complete culture medium supplemented with 10% fetal bovine serum (FBS) and 1% antibiotic–antimycotic solution. Upon reaching approximately 80–90% confluence, cells were detached using 0.25% trypsin–EDTA solution (Sigma-Aldrich, St. Louis, MO, USA). Prior to trypsinization, the culture medium was removed, and cells were gently rinsed with phosphate-buffered saline (PBS, Sigma-Aldrich, USA). Following detachment, 5 mL of cell suspension was transferred to 5 mL centrifuge tubes and centrifuged at 1200 rpm for 10 min (Thermo Fisher Scientific, Waltham, MA, USA). The supernatant was discarded, and the cell pellet was resuspended in 1 mL of complete culture medium.

##### Cell Counting and Seeding

Cell concentration was determined using a Countess 3 Automated Cell Counter (Thermo Fisher Scientific, Waltham, MA, USA). Based on the obtained counts, cell suspensions were adjusted to achieve a final concentration allowing for seeding 25,000 cells in 100 µL per insert well. Cells were seeded into silicone 3-well inserts placed in 12-well plates, creating a defined physical barrier that prevented cell migration into the central area. Each experimental condition was performed in triplicate. Control groups (without GO nanofilm exposure) were included for both cell lines. After seeding, cells were incubated for 24 h under standard culture conditions to allow for attachment and formation of a confluent monolayer.

##### Wound Creation and Migration Assay

After 24 h, silicone inserts were carefully removed to generate a uniform cell-free gap (wound area). Wells were gently washed with PBS to remove detached cells, and fresh culture medium containing reduced serum concentration (2% FBS) was added to minimize cell proliferation and ensure that wound closure resulted primarily from cell migration rather than cell division. Subsequent images were acquired at 24 h and 72 h to monitor wound closure over a total observation period of 72 h. All measurements were performed in triplicate.

##### Image Analysis

Cell migration was quantified by measuring the wound area at each time point using ImageJ software ver. 1.54 (NIH, Bethesda, MD, USA). The percentage of wound closure was calculated according to the formula:

$$\mathrm{Wound}\;\mathrm{Closure}\;(\%)\;=\;\frac{A0-At}{A0}\times 100$$

where A0 represents the initial wound area at 24 h and At represents the wound area at the respective time point (72 h). All experiments were performed in three independent biological replicates. Results were expressed as mean ± standard deviation (SD).

#### 4.3.6. Total RNA Extraction and Real-Time PCR

Gene expression analysis in pancreatic cancer cells of the BxPC-3 and AsPC-1 lines was performed using the RT-qPCR (Reverse Transcriptase—Quantitative Polymerase Chain Reaction) method, with three biological replicates and four technical replicates per sample.

##### RNA Extraction and cDNA Synthesis

Total RNA was isolated using a PureLink^®^ RNA Mini Kit (Ambion™ Life Technologies, Foster City, CA, USA). The cell pellets obtained from the BxPC-3 and AsPC-1 cell lines were resuspended in a lysis buffer with the addition of 1% 2-mercaptoethanol, and the pellets were homogenized in a TissueLyser ball mill (Qiagen, Germantown, MD, USA) for 2 × 5 min at 50 Hz. The probes centrifuged at 12,000× *g*, and the pellets were discarded. The supernatant containing total RNA was transferred to a new clean tube and processed according to the manufacturer’s instructions. The RNA samples were eluted in 50 µL RNase-free water and stored at −80 °C until use. The RNA concentration was measured using a NanoDrop 2000 spectrophotometer (Thermo Scientific, Wilmington, DE, USA). The cDNA was synthesized with a cDNA High-Capacity Reverse Transcription Kit (Applied Biosystems, Foster City, CA, USA) to reverse-transcribe the mRNA, using 2200 ng per reaction. The concentration of obtained cDNA was measured using a NanoDrop 2000 spectrophotometer and stored for further analysis at −20 °C.

#### 4.3.7. Quantitative Real-Time PCR

The reaction was carried out in 96-well plates using the Luminaris Color HiGreen reagents and the Luminaris Color HiGreen qPCR Master Mix (Thermo Fisher Scientific); 100 ng of cDNA was used for each reaction. The following genes were examined: *IL-8*, *mTOR*, *PI3KCA*, *PI3KCB*, *PCNA*, *AKT1*, *PCNA*, *β-catenin*, *Vim2* and *humanFAK*. The primers used for this procedure are presented in Table 1. Glyceraldehyde-3-phosphate dehydrogenase (*GAPDH*) was used as the reference housekeeping gene. The reaction conditions were set as specified by the manufacturer, and each sample was analyzed in duplicate. The procedure was conducted using a StepOnePlus™ Real-Time PCR System.

#### 4.3.8. Statistical Analysis

Statistical analysis and graphs were performed using GraphPad Prism 8.0.2 (GraphPad Software Inc., San Diego, CA, USA). Student’s *t*-test was used to analyze gene expression results (RT-qPCR). To assess cancer cell metabolic activity (XTT test), one-way analysis of variance (ANOVA) and Tukey’s post hoc test were used. Results were considered statistically significant at *p*-value < 0.05.

## 5. Conclusions

The current study shows that GO, applied in aerosol form to create a surface-bound nanofilm, influences the biological behavior of PADC cells in vitro. The findings indicate that the GO aerosol nanofilm primarily acts as a mechanobiologically active surface rather than as a conventional cytotoxic nanoparticle. By altering nanoscale topography and adhesion conditions, the nanofilm disrupts cytoskeletal organization, cell spreading, and migration, thereby reducing viability and impairing wound-healing capacity. The observed transcriptional patterns suggest that GO aerosol-induced surface modifications may impact proliferation and EMT-related signaling pathways, depending on the cell line. Importantly, the use of an aerosol-derived GO nanofilm distinguishes this approach from classical suspension-based exposure models and underscores the potential of surface-engineering strategies to modulate tumor cell behavior via microenvironmental cues. The modulation of adhesion and migration driven by mechanical factors may be relevant for reducing residual tumor cell activity and preventing metastatic dissemination. This concept should be evaluated in future in vivo studies. However, the current research provides evidence that GO aerosol nanofilms could be a promising platform for strategies focused on the microenvironment in pancreatic cancer research.

## Figures and Tables

**Table 1:** Primers sequences of used genes.

Gene	Sequence 5′ à 3′
*GAPDH* (*ref*)	*F:* GTCTCCTCTGACTTCAACAGCG *R:* ACCACCCTGTTGCTGTAGCCAA
*IL-8*	*F:* GAGAGTGATTGAGAGTGGACCAC *R:* CACAACCCTCTGCACCCAGTTT
*PCNA*	*F:* CAAGTAATGTCGATAAAGAGGAGG *R:* GTGTCACCGTTGAAGAGAGTGG
*mTOR*	*F:* AGCATCGGATGCTTAGGAGTGG *R:* CAGCCAGTCATCTTTGGAGACC
*PI3KCA*	*F:* GAAGCACCTGAATAGGCAAGTCG *R:* GAGCATCCATGAAATCTGGTCGC
*PI3KCB*	*F:* GGTAATCGGAGGATAGGGCAGT *R:* CGGCAGTATGCTTCAAGGATGAC
*AKT1*	*F:* TGGACTACCTGCACTCGGAGAA *R:* GTGCCGCAAAAGGTCTTCATGG
*N-cadherin*	*F:* CCTCCAGAGTTTACTGCCATGAC *R:* GTAGGATCTCCGCCACTGATTC
*β-catenin*	*F*: CCTATGCAGGGGTGGTCAAC *R*: CGACCTGGAAAACGCCATCA
*Vim2*	*F*: CCTCACCTGTGAAGTGGATGC *R*: CAACGGCAAAGTTCTCTTCCA
*humanFAK*	*F*: CCCACCAGAGGAGTATGTCC *R*: CCCAGGTCAGAGTTCAATAG

## Data Availability

The data presented in this study are available on request from the corresponding author.

## Supplementary Materials

The following supporting information can be downloaded at: https://www.mdpi.com/article/10.3390/ijms27104341/s1.
