# Supplementary material for: Aerosol-Derived Graphene Oxide Nanofilm Suppresses Adhesion-Dependent Survival and Migration in Pancreatic Ductal Adenocarcinoma Cells
Source: Int J Mol Sci. 2026 May 13;27(10):4341. doi: 10.3390/ijms27104341 (PMC13207085; doi:10.3390/ijms27104341)
Supplement: Supplementary file 1 [file ijms-27-04341-s001.zip › ijms-4296040-supplementary.pdf]

*Supplementary information*

# Aerosol-derived graphene oxide nanofilm suppresses adhesion-dependent survival and migration in pancreatic ductal adenocarcinoma cells

**Aleksandra Ciechońska [1], Mateusz Wierzbicki [2], Barbara Nasiłowska [3], Barbara Wójcik [2], Wojciech Skrzeczanowski [3], Katarzyna Ziółkowska [4] and Marta Kutwin [2,6,*]**

[1] Faculty of Animal Breeding, Bioengineering and Conservation, Warsaw University of Life Sciences, 02-786 Warsaw, Poland; s213007@sggw.edu.pl
[2] Institute of Biology, Department of Nanobiotechnology, Warsaw University of Life Sciences, 02-786 Warsaw, Poland; mateusz_wierzbicki@sggw.edu.pl (M.W.); barbara_wojcik1@sggw.edu.pl (B.W.)
[3] Biomedical Engineering Centre, Institute of Optoelectronics, Warsaw Military University of Technology, 00-908 Warsaw, Poland; barbara.nasilowska@wat.edu.pl (B.N.); wojciech.skrzeczanowski@wat.edu.pl (W.S.)
[4] Faculty of Biology and Biotechnology, Warsaw University of Life Sciences, 02-776 Warsaw, Poland; s215129@sggw.edu.pl
* Correspondence: marta_kutwin@sggw.edu.pl

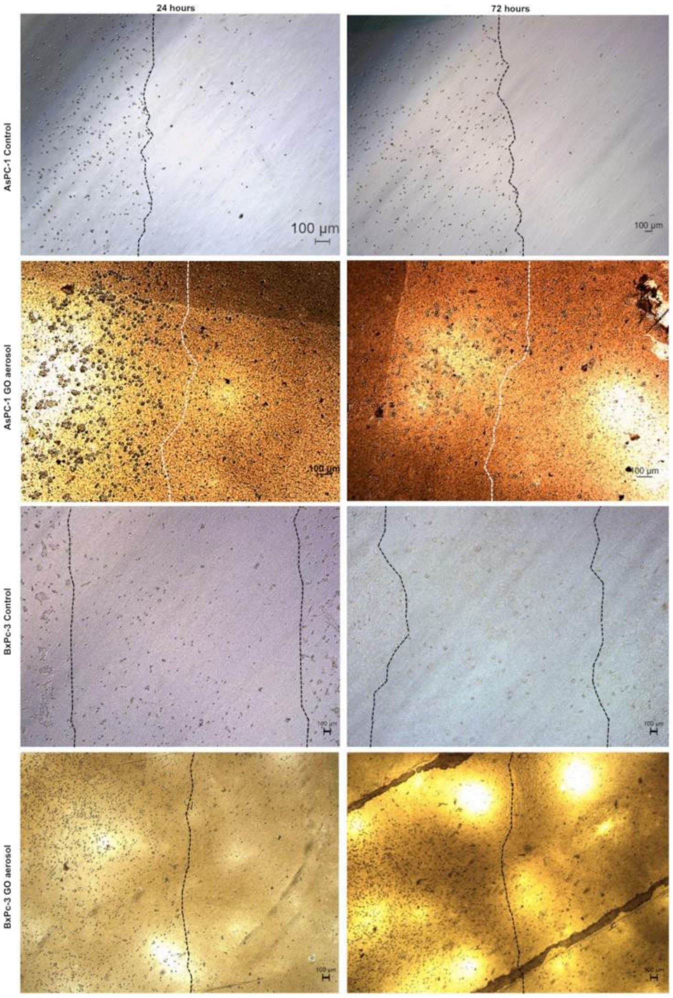


**Figure S1.** Representative optical microscopy images of wound healing assay performed on AsPC-1 and BxPC-3 pancreatic cancer cell lines cultured under control conditions and on GO aerosol-derived nanofilm at 24 h and 72 h. Dashed lines mark the wound edges used for migration analysis. Scale bars: 100 μm.
